# Supplementary material for: Individual and community factors determining delayed leprosy case detection: A systematic review
Source: PLoS Negl Trop Dis. 2021 Aug 12;15(8):e0009651. doi: 10.1371/journal.pntd.0009651 (PMC8360380; doi:10.1371/journal.pntd.0009651)
Supplement: S1 Text — (DOCX) [file pntd.0009651.s002.docx]

SI Text – Search strategy for each database

**Embase.com**

('case finding'/de OR 'delayed diagnosis'/de OR 'therapy delay'/exp OR 'time to treatment'/de OR (('leprosy'/exp/dm_di OR diagnosis/de) AND ('social aspect'/de OR 'stigma'/de OR 'symptomatology'/de OR 'symptom'/de OR 'knowledge'/de OR age/de OR 'fear'/exp OR emotion/de OR 'social isolation'/exp OR 'travel'/de OR 'social behavior'/exp OR 'independence'/de OR 'social status'/exp OR 'gender and sex'/de OR gender/de OR 'health care personnel'/exp OR 'personal experience'/de OR 'patient attitude'/exp OR 'help seeking behavior'/exp OR 'motivation'/de OR 'socioeconomics'/exp OR 'cultural factor'/de OR 'sex difference'/de)) OR (((case OR lepra OR leprosy) NEAR/3 (finding OR detect*)) OR ((delay* OR missed OR early OR earlier OR time-to) NEAR/6 (diagnos* OR detect* OR therap* OR treat*)) OR (early NEAR/3 present*) OR ((lepr* NEAR/6 diagno*) AND (social* OR travel* OR determinant* OR knowledge OR ((age OR associate* OR contribut* OR prevent*) NEAR/3 factor*) OR stigma* OR sociocultur* OR socio* OR socioeconom* OR jeopard* OR dilemma* OR polic* OR exclusion* OR complian* OR noncomplian* OR adher* OR nonadher* OR attitude* OR seeking OR motiv* OR deterring OR deterrant* OR cultur* OR dependence OR low*-status OR gender* OR promoting OR education* OR campaign*))):ab,ti) AND ('leprosy'/exp OR (leprosy OR Hansen OR lepra*):ab,ti)

**Medline All Ovid**

(Delayed Diagnosis/ OR Time-to-Treatment/ OR ((Leprosy/di OR Diagnosis/) AND (Social Stigma/ OR Knowledge/ OR Age Factors/ OR Fear/ OR exp Emotions/ OR Social Isolation/ OR Social Desirability/ OR Social Class/ OR Travel/ OR Social Behavior/ OR Sex/ OR exp Health Personnel/ OR Attitude/ OR Attitude to Health/ OR Help-Seeking Behavior/ OR Motivation/ OR Socioeconomic Factors/ OR Culture/)) OR (((case OR lepra OR leprosy) ADJ3 (finding OR detect*)) OR ((delay* OR missed OR early OR earlier OR time-to) ADJ6 (diagnos* OR detect* OR therap* OR treat*)) OR (early ADJ3 present*) OR ((lepr* ADJ6 diagno*) AND (social* OR travel* OR determinant* OR knowledge OR ((age OR associate* OR contribut* OR prevent*) ADJ3 factor*) OR stigma* OR sociocultur* OR socio* OR socioeconom* OR jeopard* OR dilemma* OR polic* OR exclusion* OR complian* OR noncomplian* OR adher* OR nonadher* OR attitude* OR seeking OR motiv* OR deterring OR deterrant* OR cultur* OR dependence OR low*-status OR gender* OR promoting OR education* OR campaign*))).ab,ti.) AND (Leprosy/ OR (leprosy OR Hansen OR lepra*).ab,ti.)

**Web of Science**

TS=(((((case OR lepra OR leprosy) NEAR/2 (finding OR detect*)) OR ((delay* OR missed OR early OR earlier OR time-to) NEAR/5 (diagnos* OR detect* OR therap* OR treat*)) OR (early NEAR/2 present*) OR ((lepr* NEAR/5 diagno*) AND (social* OR travel* OR determinant* OR knowledge OR ((age OR associate* OR contribut* OR prevent*) NEAR/2 factor*) OR stigma* OR sociocultur* OR socio* OR socioeconom* OR jeopard* OR dilemma* OR polic* OR exclusion* OR complian* OR noncomplian* OR adher* OR nonadher* OR attitude* OR seeking OR motiv* OR deterring OR deterrant* OR cultur* OR dependence OR low*-status OR gender* OR promoting OR education* OR campaign*)))) AND ((leprosy OR Hansen OR lepra*)))

**Cochrane CENTRAL**

((((case OR lepra OR leprosy) NEAR/3 (finding OR detect*)) OR ((delay* OR missed OR early OR earlier OR time-to) NEAR/6 (diagnos* OR detect* OR therap* OR treat*)) OR (early NEAR/3 present*) OR ((lepr* NEAR/6 diagno*) AND (social* OR travel* OR determinant* OR knowledge OR ((age OR associate* OR contribut* OR prevent*) NEAR/3 factor*) OR stigma* OR sociocultur* OR socio* OR socioeconom* OR jeopard* OR dilemma* OR polic* OR exclusion* OR complian* OR noncomplian* OR adher* OR nonadher* OR attitude* OR seeking OR motiv* OR deterring OR deterrant* OR cultur* OR dependence OR low* NEXT status OR gender* OR promoting OR education* OR campaign*))):ab,ti) AND ((leprosy OR Hansen OR lepra*):ab,ti)

**WHO Global Health Library**

(lepr* OR hansen*) AND (((diagnos* OR therap* OR treatment*) AND (delay*)) OR "time-to treatment" OR "time-to diagnosis" OR (diagnos* AND (social* OR stigma)) OR (case-finding))
