# Supplementary material for: Individual and community factors determining delayed leprosy case detection: A systematic review
Source: PLoS Negl Trop Dis. 2021 Aug 12;15(8):e0009651. doi: 10.1371/journal.pntd.0009651 (PMC8360380; doi:10.1371/journal.pntd.0009651)
Supplement: Attachment — Submitted filename: comments late diagnosis_Dharmawan et al, May 2021.pdf [file pntd.0009651.s003.pdf]

Individual and community factors determining delayed leprosy case detection: A systematic review

Dear Editor,

Thank you for the opportunity to review this interesting paper submitted by Dharmawan et al. Late diagnosis is a particularly important issue for leprosy control and this systematic review brings interesting data.

Please find few comments and suggestions bellow:

Abstract and Author summary

Lines 29 and 54 - We recommend specifying the last month included for publications from 2021. The text, as it reads now, suggests that the whole year was included which is not possible.

Introduction

Line 81 - Prevalence rate was not cited previously. We recommend including a brief comment in the last paragraph or to exclude “prevalence rate” from this phrase, maintaining only the incidence rate here.

Line 82/83 - It is possible that someone diagnosed with leprosy may have visible deformities from other causes, as genetic malformations, or from accidents. Thus, we suggest rephrasing to: “defined as people with visible deformities due to leprosy neuropathy.”

Lines 85/86 - We suggest including the relative percentages of G2D among new cases detected in each country or the G2D rate per million inhabitants, to allow comparation among them.

Line 93 - Prevalence should be defined earlier, soon after its first insertion in the text.

Results

Line 258 - Lower incidence of leprosy or G2D?

Line 262 – This is the first time the acronym MB is used, please use the entire word.

Lines 282/283 – “sharing a house with a person affected by leprosy” as a factor associated with delayed diagnosis is apparently controversial, but this factor was not included in your discussion.

Line 312 – “Reduced delay” means earlier diagnosis. Is this correct?

Discussion

Lines 327-329 – “A linear correlation between delayed case detection and the percentage of G2D indicated that the longer the delay, the more common and the greater the severity.”

Comment – Although the severity of G2D can be measured by the EHF score (eyes, hands and feet), this scale of severity was not discussed in this review.

Lines 337/338 – “Another highlight of this review is that delayed case detection is closely related to health-service-seeking behavior.”

Comment - This was just cited on page 17 lines 329-330. The term “another” does not seem adequate here.

Lines 347-379 – “beliefs that leprosy is caused by a curse or a spirit or other supernatural cause may also cause people who have early signs of leprosy *not to seek timely treatment* at qualified healthcare services.”

Comment - It was said that this was associated with a “reduced delay” (see line 312). Please check the right meaning of this expression.

Line 352 – “Stigma and the fear of it“… - This seems repeated with last sentence. We suggest rewriting lines 350-353 as a single phrase.

Lines 374/375 – “It can be prevented by early detection, thereby contributing considerably to reductions in disease burden.”

Comment – We suggest including “adequate treatment” once leprosy reactions and neuritis may lead to disabilities as well, even in early detected cases.

Lines 410-417 - There are huge differences among national programs from different countries and certainly not all TB national programs adopt these approaches. We suggest removing this whole paragraph once it does not seem to contribute significantly to the discussion and may cause some confusion.

Line 422 - We suggest replacing “association” by “correlation” once your analysis allows this conclusion.

Line 427 - We suggest considering possible loss of information from Brazilian papers published in Portuguese as well, once this is a highly endemic area.

Figures

Figure 4 is remarkable and brings new light for this subject.

From our perspective, data from figure 2 could be better presented as a table together with their respective confident intervals.
