# Supplementary material for: Individual and community factors determining delayed leprosy case detection: A systematic review
Source: PLoS Negl Trop Dis. 2021 Aug 12;15(8):e0009651. doi: 10.1371/journal.pntd.0009651 (PMC8360380; doi:10.1371/journal.pntd.0009651)
Supplement: Attachment — Submitted filename: Comments_Plos.docx [file pntd.0009651.s004.docx]

Background: the focus is confused because includes the PD as the expression of delay in diagnosis.

- The number of new leprosy cases is declining globally, but the disability caused by
- 20  leprosy remains an important disease burden. The chance of disability is increased by delayed
- 21  case detection.

To identify determinants of delayed detection or Grade 2 Disability (G2D),

Conclusion - Delayed leprosy case detection is clearly correlated with increased disability (it was not the main aim?) the authors assumed this assumption at the begging.

Method

- the period of delay
- and (b) the occurrence of Grade 2 Disability (G2D

- English between January 1, 2000 and January 31,
- 125  2021.  Why not in Spanish or Portuguese?

Repeated

- To select articles for full-text screening, two reviewers (YD and AF) independently screened article
- 129  titles and abstracts. Data from articles were extracted and double-entered into Microsoft Excel:
- 130  two reviewers (YD and AF) independently screened article titles and abstracts and then extracted
- 131  the selected articles and double-entered these into Microsoft Excel.
- One [29] of three studies investigating the role of geographic area [27, 29, 31] reported a delay in
- 320  case detection that was shorter (21 months) in a non-endemic area than in an endemic area (23
- 321  months) (p<0.01).

It is the opposite in LEM.
