# Supplementary material for: Individual and community factors determining delayed leprosy case detection: A systematic review
Source: PLoS Negl Trop Dis. 2021 Aug 12;15(8):e0009651. doi: 10.1371/journal.pntd.0009651 (PMC8360380; doi:10.1371/journal.pntd.0009651)
Supplement: Attachment — Submitted filename: Reply to Reviewer Comments_PNTD-D-21-00581_final_18062021.docx [file pntd.0009651.s005.docx]

Dear Dr. Alberto Novaes Ramos Jr,

Associate Editor

PLOS Neglected Tropical Diseases

Dear Dr. Kristien Verdonck

Deputy Editor

PLOS Neglected Tropical Diseases

On behalf of all co-authors, I thank you for your response and the reviewer’s comments on our manuscript entitled ‘Individual and community factors of delayed leprosy case detection: A systematic review.’

We have revised our manuscript according to the comments of the reviewers, visible as track changes. Here we give a point-by-point reply to the reviewer’s comments:

# Abstract and Author summary

**Reviewer #1**

*Comment 1*

**Lines 29 and 54** - We recommend specifying the last month included for publications from 2021. The text, as it reads now, suggests that the whole year was included which is not possible.

*Reply:*

We have revised it by adding the full dates of the period.

Line 29: had been published between January 1, 2000, and January 31, 2021

Line 54: studies in our analysis, published between January 1, 2000, and January 31, 2021

**Reviewer #3**

*Comment 1*

Background: the focus is confused because includes the PD as the expression of delay in diagnosis.

Line 20: The number of new leprosy cases is declining globally, but the disability caused by leprosy remains an important disease burden. The chance of disability is increased by delayed case detection.

*Reply:*

If we understand this comment correctly, the reviewer refers to the fact that delayed case detection can have many causes and that our review is not focused on all these causes. We have therefore adapted the sentence as follows:
Line 21: This review focuses on the individual and community determinants of delayed leprosy case detection.

*Comment 2*

To identify determinants of delayed detection or Grade 2 Disability (G2D),

*Reply:*

Here we understand the confusion that we have mistakenly introduced. We have therefore deleted “or Grade 2 Disability (G2D)” from the text (lines 25)

# Introduction

**Reviewer #1**

*Comment 2*

**Line 81** - Prevalence rate was not cited previously. We recommend including a brief comment in the last paragraph or to exclude “prevalence rate” from this phrase, maintaining only the incidence rate here.

*Reply:*

We have deleted “prevalence” from the text.

*Comment 3*

**Line 82/83** - It is possible that someone diagnosed with leprosy may have visible deformities from other causes, as genetic malformations, or from accidents. Thus, we suggest rephrasing to: “defined as people with visible deformities due to leprosy neuropathy.”

*Reply:*

We thank the reviewer for this suggestion and have revised the manuscript accordingly.

Line 82-84 now reads:

Another important indicator of the burden of disease beside incidence rates is the number of new cases with Grade 2 Disability (G2D), which are defined as people with leprosy who have visible deformities due to leprosy neuropathy.

*Comment 4*

**Lines 85/86** - We suggest including the relative percentages of G2D among new cases detected in each country or the G2D rate per million inhabitants, to allow comparation among them.

*Reply:*

We have revised it by adding the percentages in the text.

Line 84-87:

Although the worldwide percentage of new cases with G2D fell slightly from 5.8% in 2010 to 5.3% in 2019, the percentage of new cases with G2D was higher in 2019 in Brazil (8.4%), Ethiopia (12.8%), Nepal (6.6%), and Nigeria (15.2%) than in 2018 (7.4%, 8.0%, 4.1%, and 14.6%, respectively) [2].

*Comment 5*

**Line 93** - Prevalence should be defined earlier, soon after its first insertion in the text.

*Reply:*

We have deleted the term prevalence earlier, so we leave the text here as it is.

# Methods

**Reviewer #1:**

Proceed without modifications

*Reply not needed*

**Reviewer #2:**

*Comment 1*

The objectives of the study are clearly articulated with a clear testable hypothesis stated.

*Reply not needed*

*Comment 2*

The study design is appropriate to address the stated objectives.

*Reply not needed*

*Comment 3*

Since the study is a systematic review about individual and community factors determining delayed leprosy case detection, the population, sample size, statistical analysis are not involved in the study.

*Reply not needed*

*Comment 4*

The methods for systematic review in the study is proper.

*Reply not needed*

**Reviewer #3:**

*Comment 3*

In the background the authors mentioned the increased chance of disability by delayed case detection. At the same time, they used physical disability as a measure of delay and analyzed this factor (G2). In the conclusion, they affirmed “Delayed leprosy case detection is clearly correlated with increased disability”. To maintain this conclusion the aim has to be changed to confirm this issue.

*Reply:*

As demonstrated in literature previously, we consider G2D as an indicator (not measure) of delay (line 94). We analyze its correlation (or association) with delay. With our analysis, we confirm that increased detection delay is correlated with and increased prevalence of G2D, which was indeed described previously in the literature (see reference 7).

*Comment 4*

See in Method***: Repeated*** - To select articles for full-text screening, two reviewers (YD and AF) independently screened article titles and abstracts. Data from articles were extracted and double-entered into Microsoft Excel: two reviewers (YD and AF) independently screened article titles and abstracts and then extracted the selected articles and double-entered these into Microsoft Excel.

Lines 129-131: To select articles for full-text screening, two reviewers (YD and AF) independently screened article titles and abstracts. Data from articles were extracted and double-entered into Microsoft Excel: two reviewers (YD and AF) independently screened article titles and abstracts and then extracted the selected articles and double-entered these into Microsoft Excel.

*Reply:*

Indeed! We have removed the repeated sentences from the manuscript (line 130-131).

*Comment 5*

… (a) the period of delay and (b) the occurrence of Grade 2 Disability (G2D)

*Reply:*

We use both terms to search for delayed case detection in our systematic review because some papers mention delayed case detection expressed in duration, and others use the occurrence of the G2D as an indicator of delayed case detection. We have adjusted the text as follows:

Lines 120-122: “In this systematic review, we searched for delayed leprosy case detection based on (a) the period of delay calculated from the beginning of signs or symptoms to diagnosis, either in numerical or categorical values; and (b) the occurrence of Grade 2 Disability (G2D).“

*Comment 6*

English between January 1, 2000 and January 31; Why not in Spanish or Portuguese?

*Reply:*

Unfortunately, we cannot read or write Spanish and Portuguese and therefore are unable to assess publications in these (and many other) languages from leprosy endemic countries. In the study protocol registered in PROSPERO this was mentioned and we now also mention it as a research limitation in the text.

Lines 438-440:

Second, as we included only literature in English, we could not capture publications in other languages that may have originated in countries with a high leprosy burden, such as Indonesia and Brazil

# Results

**Reviewer #1:**

*Comment 6*

**Line 258** - Lower incidence of leprosy or G2D?

*Reply:*

Lower incidence of G2D. We have revised it in the text.

Lines 259-261:

Three [18, 19, 22] of the ten studies investigating the role of sex [14, 18-22, 25, 27, 28, 33] reported a lower incidence of G2D among females than males, and two [14, 27] reported shorter delay in detection among females.

*Comment 7*

**Line 262** – This is the first time the acronym MB is used, please use the entire word.

*Reply:*

We have corrected it in the text by using the entire word “multibacillary” (line 265)

*Comment 8*

**Lines 282/283** – “sharing a house with a person affected by leprosy” as a factor associated with delayed diagnosis is apparently controversial, but this factor was not included in your discussion.

*Reply:*

In this sentence three one-off factors are recorded that were mentioned in the papers reviewed, without much further discussion. We duly record these factors in our results section but find it difficult to add further reflections to this particular finding, other than the discussion we already have on social exclusion and stigma in general.

*Comment 9*

**Line 312** – “Reduced delay” means earlier diagnosis. Is this correct?

*Reply:*

Yes, indeed, “reduced delay” means earlier diagnosis. It also means “shorter delay”. We have revised the text by replacing “Reduced delay” with “shorter delay”

Line 325:

One [25] of four studies that investigated social values [25,32,37,38] found that shorter delay was…

**Reviewer #2:**

*Comment 5*

The analysis presented matches the analysis plan and the results are clearly and completely presented.

*Reply not needed*

**Reviewer #3:**

*Comment 7*

One [29] of three studies investigating the role of geographic area [27, 29, 31] reported a delay in case detection that was shorter (21 months) in a non-endemic area than in an endemic area (23 months) (p<0.01). It is the opposite in LEM.

*Reply:*

Indeed, this was reported in a study conducted in China in 2000. We will address LEM further below.

# Discussion

**Reviewer #1:**

*Comment 10*

**Lines 327-329** – “A linear correlation between delayed case detection and the percentage of G2D indicated that the longer the delay, the more common and the greater the severity.”

Comment – Although the severity of G2D can be measured by the EHF score (eyes, hands and feet), this scale of severity was not discussed in this review.

*Reply:*

We have revised it by adding more information on the EHF in the discussion and explaining how the EHF score could help our understanding of the correlation between detection delay and disability, and why we did not use the EHF score.

Lines 443-450:

Fourth, the WHO leprosy disability grading system grades patients according to the presence of disabilities of the eyes, hands, and feet [4, 68]. G2D is usually reported to WHO as the proportion of people with G2D at any body site among leprosy cases newly diagnosed in a specific year. The sum score of these six body sites is called the Eye-Hand-Foot (EHF) score and is used as an overall indicator of the impairment status of an individual with leprosy [68]. Taking EHF scores into account instead of G2D could provide a more nuanced insight into the correlation between detection delay and level of disability. Unfortunately, EHF scores are not widely available.

*Comment 11*

**Lines 337/338** – “Another highlight of this review is that delayed case detection is closely related to health-service-seeking behavior.”

Comment - This was just cited on page 17 lines 329-330. The term “another” does not seem adequate here.

*Reply:*

We have revised it. “Another” is changed to “A”

Lines 352-353:

A highlight of this review is that delayed case detection is closely related to health-service-seeking behavior, i.e., seeking care from qualified healthcare facilities in a timely way.

*Comment 12*

**Lines 347-379** – “beliefs that leprosy is caused by a curse or a spirit or other supernatural cause may also cause people who have early signs of leprosy not to seek timely treatment at qualified healthcare services.”

Comment - It was said that this was associated with a “reduced delay” (see line 312). Please check the right meaning of this expression.

*Reply:*

We have revised this sentence in the text, replacing the term “reduced” by “shorter”. (line 325). Only one paper described supernatural beliefs as cause for a shorter detection delay, but other papers observe that such beliefs contribute to a longer detection delay. This was mentioned in the discussion, but it was not clear from our text in the results section. We have revised the sentence as follows:

One [25] of four studies that investigated social values [25,32,37,38] found that shorter delay was associated with a belief that leprosy was caused by a curse, spirit, or ghost (OR=0.28; 95% CI=0.08-0.97]), but one other study indicated that these kinds of beliefs led to longer delay [38]. (lines 325-327)

*Comment 13*

**Line 352** – “Stigma and the fear of it“…

This seems repeated with last sentence. We suggest rewriting lines 350-353 as a single phrase.

*Reply:*

We have revised this sentence in the text. We have brought the two sentences together in the manuscript Lines 365-367:

Stigma and the fear of it can lead people with leprosy to conceal their condition, or to visit a distant health center in order to avoid being recognized by people from their community, and could thus cause delayed case detection

*Comment 14*

**Lines 374/375** – “It can be prevented by early detection, thereby contributing considerably to reductions in disease burden.”

Comment – We suggest including “adequate treatment” once leprosy reactions and neuritis may lead to disabilities as well, even in early detected cases.

*Reply:*

We have revised the sentence by adding “adequate treatment”

Lines 388-390:

It can be prevented by early detection and adequate treatment, thereby contributing considerably to reductions in disease burden

*Comment 15*

**Lines 410-417** - There are huge differences among national programs from different countries and certainly not all TB national programs adopt these approaches.

We suggest removing this whole paragraph once it does not seem to contribute significantly to the discussion and may cause some confusion.

*Reply:*

We prefer to keep this paragraph, but we have changed the word ‘similar’ to ‘comparable’ to describe the pathway of health-seeking behavior in leprosy is comparable within Tuberculosis (lines 423-424). We have also made some other edits to the text to avoid the impression that control programs are always similar (lines 425-430).

*Comment 16*

**Line 422** - We suggest replacing “association” by “correlation” once your analysis allows this conclusion

*Reply:*

We have replaced “association” by “correlation”

Line 435:

.. confirms the strong linear correlation between delay and G2D.

*Comment 17*

**Line 427** - We suggest considering possible loss of information from Brazilian papers published in Portuguese as well, once this is a highly endemic area.

*Reply:*

We have included Brazil as the country with the largest number of cases with G2D. Indeed, we had no access to publications from Brazil in Portuguese to include in this review. This is also the case for publications in the mother tongue of other leprosy endemic countries. It has been mentioned as a limitation of this study (lines 438-440).

# Conclusions

**Reviewer #1:**

Please find all comments and recommendations as an attached document

*Reply not needed*

**Reviewer #2:**

The conclusions are supported by the data presented. The limitations of analysis are clearly described. Public health relevance is addressed.

*Reply not needed*

**Reviewer #3:**

*Comment 8*

The aim is “To identify determinants of delayed detection or Grade 2 Disability (G2D),” It means that the Grade 2 Disability is assumed as the same (the expression) of delayed detection. This way, it cannot be the conclusion.

*Reply:*

See our reply to this earlier on this point under methods. For further clarification, we have revised this sentence in the conclusion.

Line 457-458: This review confirms that delayed case detection is clearly correlated with increased disability in leprosy, and therefore its reduction should be a priority of leprosy programs.

*Comment 9*

Conclusion - Delayed leprosy case detection is clearly correlated with increased disability (it was not the main aim?) the authors assumed this assumption at the begging.

*Reply:*

We have revised this sentence in the conclusion (see above).

# Editorial and Data Presentation Modifications?

**Reviewer #1:**

Please find all comments and recommendations as an attached document

*Reply not needed*

**Reviewer #3:**

*Comment 10*

The authors mentioned the limitations for the use of only publications in English. However, they do not mention the reasons why the results of Leprosy Monitoring Evaluation (LEM) usually publicized in English were not used. In the LEM reports the time between the sings and symptoms until the diagnosed is one of the most important point of the analyses. The results usually are the opposite of the findings: “ One [29] of three studies investigating the role of geographic area [27, 29, 31] reported a delay in case detection that was shorter (21 months) in a non-endemic area than in an endemic area (23 months) (p<0.01).

*Reply:*

Our extensive systematic literature search did not reveal any results relating to the Leprosy Elimination Monitoring (LEM). The guideline for this activity was published by the WHO in the year 2000 and indeed includes the indicator ‘average detection delay’. We cannot evaluate the quality of the original data ourselves and we have not come across peer-reviewed research articles with quality evaluations of this data by others. From a scientific perspective data on detection delay from LEM do not provide a basis for inclusion in our systematic review.

*Comment 11*

Usually the less endemic more delay of diagnosis explained by the centralization of services of leprosy diagnosis - it is the rational for rare diseases when the doctors are not prepared to identify the signs and symptoms. Some individual and community factors were explained by the stigmatization of the disease. The stigma is known a problem for leprosy control. However, the social vulnerability resulted by social exclusion, poverty, illiteracy, difficulties to access of health services are different of stigma. Considering leprosy as a neglected tropical disease the authors should discuss this problem independently of stigma.

*Reply:*

The reviewer is perfectly correct in stating that social vulnerability by social exclusion, poverty, illiteracy, and difficulties to access of health services are different of stigma. In the paragraph of the discussion where stigma is mentioned, we first dwell on several personal and community related factors of detection delay, of which stigma is certainly one important aspect. Another important set of factors related to detection delay have to do with health services, including the ability of doctors and other health staff to recognize rare and neglected diseases of poverty. Health service-related factors of detection delay will be the topic of a second systematic review we are preparing for publication.

# Summary and General Comments

**Reviewer #1:**

Please find all comments and recommendations as an attached document

*Reply not needed*

**Reviewer #2:**

If all factors related to individual and community were presented in one table or several figures, it would be easy to read for readers since it only described the reviewed papers.

*Reply:*

We prefer not to add an additional table or figure to capture all factors to avoid duplication of information. However, we have added subheadings under the headings “Individual Factors”, “Socioeconomic factors” and “Social and community factors” in the results section in order to bring out the different factors more explicitly.

**Reviewer #3:**

The absence of the LEM results carried out in the endemic countries for many rounds should be justified.

*Reply:*

See our reply in comment 10 above.

# Figures

**Reviewer #1:**

Figure 4 is remarkable and brings new light for this subject.

From our perspective, data from figure 2 could be better presented as a table together with their respective confident intervals

*Reply:*

We believe that it is better to present the data in a figure than a table to show the range of the percentages of G2D. Furthermore, the data resulted from various study designs, so to present them with a confident interval can lead to misinterpretation. Also, not all the studies provide a confidence interval.

We hope with our revision the manuscript is now acceptable for publication in PLOS NTDs and look forward to your reply

On behalf of all co-authors, sincerely,

Yudhy Dharmawan

Y. Dharmawan, MSc

Department of Public Health

Erasmus MC, University Medical Center Rotterdam
